# Acute hemodynamic responses to high intensity interval training, moderate intensity continuous training, and vigorous intensity continuous training in active men

**DOI:** 10.3389/fphys.2026.1832895

**Published:** 2026-07-01

**Authors:** Todd A. Astorino, Andrea Michel, Hailey Schepper, Averee Wolter, Dalton Ferguson, Gabriella Ho, Michael Bauer, Matthew C. DeSouza, Cristos Birbilis, Stefano Benitez-Flores, Gregory C. Bogdanis

**Affiliations:** 1 Department of Kinesiology, California State University--San Marcos, San Marcos, CA, United States; 2 Department of Physical Education and Health, Higher Institute of Physical Education, University of the Republic, Montevideo, Uruguay; 3 School of Physical Education and Sports Science, National and Kapodistrian University of Athens, Athens, Greece

**Keywords:** intermittent exercise, oxygen consumption, oxygen transport, stroke volume, thoracic impedance

## Abstract

**Introduction:**

Cardiac output (CO), the product of heart rate and stroke volume (SV), reflects oxygen transport to skeletal muscle during exercise and is a determinant of maximal oxygen uptake (VO2max). This study assessed differences in the CO and SV response to three duration-matched sessions of cycling.

**Methods:**

Eighteen healthy non-obese men (age and VO2max = 24 ± 5 yr and 41 ± 9 mL/kg/min) initially completed incremental cycling to exhaustion to determine VO2max, maximal CO and SV, and first ventilatory threshold (VT1). Subsequent sessions included 15 min of high intensity interval training (HIIT) at workload equal to 30% above VT1, moderate intensity continuous training (MICT) at 30% below VT1, and vigorous intensity continuous training (VICT) at 10% below VT1. Pulmonary gas exchange data and thoracic impedance were used to assess changes in VO2, SV, arterio-venous oxygen difference (avO2diff), and CO during exercise.

**Results:**

Results revealed significantly higher mean VO2 in response to VICT versus HIIT (2.43 ± 0.52 vs. 2.25 ± 0.39 L/min, p = 0.016, d = 0.69) which was associated with a significantly higher (p = 0.01) avO2diff (12.4 ± 2.0 vs. 11.4 ± 1.6 mL/dL, d = 0.37), yet CO was similar (p > 0.05) between VICT and HIIT. Stroke volume was not different across protocols (p = 0.08) and displayed varied responses across participants.

**Conclusions:**

Despitelower intensity than HIIT, VICT elicited significantly higher mean VO2 and similar CO compared to HIIT, suggesting a substantial stimulus on the cardiovascular system during exercise.

## 1 Introduction

The Fick Equation demonstrates that oxygen consumption (VO_2_) is dictated by the product of heart rate (HR) and stroke volume (SV), represented as cardiac output (CO), and arteriovenous oxygen difference (avO_2_diff). Upon initiation of exercise from rest, there is an increase in VO_2_ which is due to increases in both CO and avO_2_diff (Astrand et al., 1964). However, it is apparent that maximal SV is attained at approximately 40%VO_2_max, after which HR continues to increase to further augment CO and thus VO_2_. Subsequent results from Hermansen et al. (1970) showed that men with higher VO_2_max revealed substantially higher maximal SV and in turn, CO, than less fit participants, suggesting the importance of optimizing O_2_ delivery to cause elevated values of VO_2_max. Overall, these studies established the association between SV, CO, and VO_2_max which is a fundamental concept in Exercise Physiology.

Understanding how different exercise modalities stress the cardiovascular system has important implications for optimizing aerobic training adaptations (Buchheit and Laursen, 2013). It is apparent that maintaining high fractions of %VO_2_max and/or SVmax during exercise is related to long term increases in cardiorespiratory fitness (Buchheit and Laursen, 2013). Although moderate intensity continuous training (MICT) seems to elicit maximal SV, its reliance on moderate intensities does not allow high fractions of %VO_2_max to be attained during exercise. In contrast, high intensity interval training (HIIT), brief (30 s – 4 min) repeated efforts performed at intensities up to or at VO_2_max, maximal velocity, or the workload associated with VO_2_max (Wmax), has been shown to elicit high fractions of CO/VO_2_max. Prior studies comparing HIIT with continuous exercise have generally shown higher VO_2_ and CO responses during HIIT; whereas, SV responses appear more variable across studies and participant populations (Zafeiridis et al., 2015; Falz et al., 2019; Coe and Astorino, 2024). Yet, additional studies are needed using different exercise protocols to substantiate that central O_2_ delivery, rather than O_2_ extraction, is responsible for the greater VO_2_ observed in response to HIIT.

Most previous studies examining hemodynamic responses have relied on incremental exercise or steady-state exercise protocols. Consequently, less is known about the cardiovascular response to low-volume vigorous exercise protocols commonly used in contemporary training practice, particularly vigorous intensity continuous training (VICT) and HIIT. In active men, Falz et al. (2019) compared hemodynamic responses between 4 X 4 HIIT at 90%HRmax and MICT at 70%HRmax, and results revealed significantly higher mean/peak CO and VO_2_ in response to HIIT compared to MICT, although there was no difference in SV or avO_2_diff between protocols. Zafeiridis et al. (2015) compared hemodynamic responses between three protocols having matched effort. Results showed that repeated 2 min bouts of HIIT at 90%Wmax elicit significantly higher peak CO than 29 min of vigorous intensity continuous training (VICT = 160 b/min) at 70%Wmax or repeated 30 s HIIT at 110%Wmax, although mean CO was not different between 2 min HIIT and VICT. Peak SV also did not differ across protocols, although mean SV was significantly higher in response to 2 min HIIT and VICT versus 30 s HIIT. In addition, mean VO_2_ was highest in response to VICT, and 2 min HIIT exhibited the highest peak %VO_2_max value, yet there was no significant difference in avO_2_diff across protocols. Overall, these results reveal significantly higher VO_2_ in response to HIIT compared to MICT or VICT, which is due to a higher CO rather than avO_2_diff.

Lavie et al. (2015) stated that acute changes in SV and CO may be associated with the long-term adaptive response to exercise training, emphasizing the utility of assessing these parameters in research. Results from Montero et al. (2015) in 130 participants revealed that increases in maximal CO, rather than avO_2_diff, were responsible for the increase in VO_2_max with MICT. However, these data are opposed by results in active adults (Faricier et al., 2025) showing that 12 wk of VICT led to significant increases in VO_2_max (+ 20%) which were attendant with significant increases in maximal CO (+ 15%), SV (+ 14%), and avO_2_diff (+ 4%). In inactive adults, Daussin et al. (2007) showed significant increases in VO_2_max after 8 wk of MICT which were attendant with an increase in avO_2_diff but no change in maximal CO. Nevertheless, data from a systematic review and meta-analysis of 45 studies exploring the effects of HIIT (Astorino et al., 2022) showed significant increases in maximal SV and CO were associated with a 15% increase in VO_2_max, yet five of nine studies reported a significant increase in avO_2_diff with training. Substantial variations in participant traits, exercise protocols, and methods of CO assessment may explain these disparate data and merit additional study examining the hemodynamic response to vigorous exercise.

Importantly, prior comparisons of hemodynamic responses across exercise modalities have primarily prescribed exercise intensity using %HRmax or %Wmax, which do not account for inter-individual variation in physiological thresholds. Exercise prescription relative to ventilatory threshold (VT1/GET) provides improved physiological standardization across participants (Iannetta et al., 2020). However, the respiratory compensation point (RCP/VT2) was not determined in the present study, which limits precise classification of exercise intensity domains. This study compared differences in the VO_2_, CO, SV, and avO_2_diff response to three duration-matched, low-volume regimens of cycle ergometry varying in intensity. A secondary aim was to describe the SV response to exercise, as prior studies have characterized changes in this outcome during graded exercise leading to VO_2_max rather than during continuous or interval exercise. First, it was hypothesized that compared to MICT, HIIT and VICT would elicit significantly higher VO_2_ and CO, yet no significant differences in SV or avO_2_diff would occur between bouts. Second, it was hypothesized that no significant change in SV would occur during exercise, yet varied SV responses would be demonstrated across participants. These findings may provide insight into the cardiovascular and peripheral mechanisms contributing to acute responses during vigorous exercise which may be used to develop effective and individualized exercise regimens to increase VO_2_max.

## 2 Materials and methods

Design: This study used a within subject crossover design, in which four sessions were completed at the same time of day within participants. Data were acquired from October 2024 through March 2025. All sessions were preceded by a 3 hours fast, no caffeine in the last 8 hours, and 36 h abstention from intense physical activity. In a preliminary session, VO_2_max and VT1 were determined during incremental cycling to exhaustion on an electrically-braked cycle ergometer (Velotron RacerMate, Quarq, Spearfish, SD, USA), and VT1 was used to develop intensities for the subsequent three sessions which included HIIT, VICT, and MICT. Session order was randomized across participants using randomizer.org. During all sessions, gas exchange data, hemodynamic responses, and blood lactate concentration were determined. Testing was performed in a laboratory setting with temperature and humidity equal to 20–23 °C and 40 – 60%, respectively. The project was registered with Open Science Framework (https://osf.io/dv75g).

Participants: Eighteen men completed this study, whose demographic traits are shown in **Table 1**. Inclusion criteria included BMI ≤ 30 kg/m^2^, non-smoker, healthy according to the Physical Activity Readiness Questionnaire, free of joint issues, and habitually active which was defined as participation in > 150 min/wk of physical activity in the preceding year. Written informed consent was obtained from all participants, and the research protocol was approved by the University Institutional Review Board (protocol 2190788-1).

**Table 1:** Participant physical characteristics (N = 18).

Outcome	Mean ± SD	Range
Age (yr)	24 ± 5	19 - 42
Body mass (kg)	80 ± 9	57 - 95
BMI (kg/m^2^)	25 ± 2	20.3 – 29.1
Body fat (%)	14 ± 5	5 - 22
Fat free mass (kg)	69 ± 7	53 - 83
PA (h/wk)	7 ± 2	3.5 – 11.0

BMI, body mass index; PA, physical activity.

Assessment of VO_2_max, VT1, and hemodynamic responses: Initially, height and body mass were measured using a stadiometer and balance beam scale, and percent body fat (%BF) was estimated by assessing subcutaneous fat in rotational order at the chest, abdomen, and thigh following standardized procedures for skinfolds (Jackson and Pollock, 1978). Participants began cycling at 40–70 W for 2 min followed by 20–35 W/min ramp-like increases in power output until volitional exhaustion, which was identified by pedal cadence < 50 rev/min. During exercise, participants breathed through a three-way valve, and pulmonary gas exchange data (VO_2_, VCO_2_, RER, and V_E_) were determined every 15 s using a metabolic cart (ParvoMedics TrueOne, Sandy, UT, USA), which was calibrated before exercise according to manufacturer recommendations. Due to the relatively rapid ramp rate, it is acknowledged that mean response time (MRT) effects may influence the alignment between gas exchange and workload. This limitation is addressed in the Discussion. VO_2_max was identified as the mean of the two highest consecutive values in the last 30 s of exercise, and volitional exhaustion confirmed by cadence < 50 rev/min. Maximal workload (Wmax) was identified as the power output attained at volitional exhaustion. A verification phase was not performed, which is acknowledged as a limitation. VO_2_max attainment was confirmed using RERmax ≥ 1.10 and BLa ≥ 8 mM, which was attained by all participants, and HRmax ± 10 b/min from 220-age, which was attained by 56% of participants. Blood lactate concentration (BLa) was measured 3 min post-test from a 0.7 µl blood sample via a fingertip puncture using a lancet (Owen Mumford Inc., Marietta, GA, USA) and portable analyzer (Lactate Plus, Sports Research Group, New Rochelle, NY, USA). Prior to measurement, the fingertip was cleaned with a damp paper towel, then it was dried, punctured, and the first drop of blood was wiped away. To assess the VT1, two experimenters independently examined graphs of the ventilatory equivalents of oxygen and carbon dioxide (V_E_VO_2_ and V_E_VCO_2_) versus time, using the criterion developed by Caiozzo et al. (1982) of “*the time at which V_E_VO_2_ exhibited a systematic increase without a concomitant increase in V_E_VCO_2_*.” If disagreement occurred between individuals, a third researcher was consulted. This ventilatory threshold corresponds to VT1/GET and does not represent the respiratory compensation point (RCP/VT2), which was not determined in the present study. This value was associated with power output expressed in Watt as well as a percentage of VO_2_max and HRmax.

Thoracic impedance (Physioflow Enduro, Manatec, Strasbourg, France) was performed during all sessions to estimate changes in HR, SV, and CO following established procedures (Richard et al., 2001). First, resting blood pressure was assessed in duplicate using manual sphygmomanometry at the antecubital space (Omron Tru-Gage Cuff, Omron Healthcare, Vernon Hills, IL, USA), with the average value inserted into the software during calibration of the device. Six electrodes (PhysioFlow Versa Trode, Nissha Medical Technologies, Devon, UK) were placed on each participant, two on the left supraclavicular fossa at the base of the neck, one on the right pectoralis major, one on the left rib closest to V6, and two left of the spine (on the transverse xiphoid process line and directly below). Each site was cleaned with alcohol, then an electrode gel (NuPrep; Weaver and Company, Aurora, CO, USA) was rubbed into the skin. The participant sat quietly and a 30-beat calibration procedure ensued to estimate resting HR, SV, and CO. During exercise, HR, CO, and SV were averaged every 15 seconds. Intraclass correlations for repeated estimates of SV and CO at VO_2_max in our lab from similarly-trained men performing ramp exercise on the same cycle ergometer are equal to 0.90-0.95, with no significant difference in maximal SV/CO across sessions (Astorino et al., 2015a). These results support high test-reliability results previously reported for thoracic impedance (Richard et al., 2001) in which the Authors concluded that this approach was clinically acceptable to estimate CO versus the direct Fick method. In addition, the minimum difference in CO in response to Wingate tests performed on separate days is = 1.8 L/min (Astorino et al., 2015b). Arteriovenous oxygen difference (a-vO_2_diff) was calculated as the quotient of VO_2_ (mL/min)/CO (L/min) and expressed in mL/dL.

Exercise protocols: Participants were asked to record all food and drink consumption 24 hours prior to the initial session and were encouraged to replicate this intake before the last two sessions. Upon entering the lab, participants rested for 5 min, then BLa and blood pressure were measured following procedures described above. The metabolic cart was calibrated, and six electrodes were placed on each participant to acquire continuous hemodynamic data. A 5 min warm-up at 20%Wmax preceded 15 min of exercise consisting of HIIT, MICT, or VICT during which gas exchange data were continuously acquired. Ten bouts of HIIT were performed at workload equal to 30% above VT1 using a 45 s:45 s ratio (Wen et al., 2019) of interval to recovery, with active recovery performed at 20%Wmax. MICT consisted of 15 min of cycling at workload 30% below VT1, and VICT consisted of 15 min of cycling at workload 10% below VT1. The 15 min duration was selected as preliminary work exhibited that men having similar VO_2_max as our participants frequently fatigued in response to VICT of longer durations; moreover, workload equivalent to 10% below VT1 elicited HR representative of “vigorous exercise” (Coates et al., 2023). These MICT and HIIT regimens follow protocols prescribed using VT1 (Bogdanis et al., 2021; Storey et al., 2024) and elicit ~73 and 80%HRmax and BLa = ~ 3 and 8 mM, respectively. Bouts were prescribed according to VT1 rather than %HR/VO_2_/Wmax, as these methods do not match the metabolic stress of exercise. BLa was acquired 5 min post-exercise using a fingertip blood sample, after which participants completed the 18-item Physical Activity Enjoyment scale (Kendzierski and DeCarlo, 1991).

VO_2_ and hemodynamic responses during exercise: Similar to previous studies (Zafeiridis et al., 2015; Coe and Astorino, 2024), HR, SV, CO, avO_2_diff, and VO_2_ were reported as mean and peak values for each protocol and expressed as a percentage of the maximal value derived from VO_2_max testing. Peak values were determined as the mean of the three highest consecutive 15 s values at any point of the session. Mean values were determined as the average of data from the entire session, not including pre-exercise or warmup. Values were also recorded at rest and at 3, 6, 9, 12, and 15 min of exercise, which included the average of four consecutive values acquired before each timepoint. An increase in SV was identified as a change in SV ≥ 10 mL from the 3–15 min time point, while a decrease was a decline in SV ≥ 10 mL, with no change being a < 10 mL change in this outcome from 3–15 min of exercise. Time spent > 77%HRmax was also calculated as this intensity represents vigorous exercise (Coates et al., 2023). This threshold was used descriptively in relation to public health recommendations and did not guide exercise intensity prescription. The change in HR (b/min) and VO_2_ (L/min) during each protocol was calculated as the difference in the outcome between the first and last minute of exercise.

Data analyses: Data are expressed as mean ± SD and were analyzed using SPSS Version 28.0 (IBM, Armonk, NY, USA). Normality was assessed using the Shapiro–Wilk test. Two-way repeated measures ANOVA was used to compare differences in each outcome across time (6 levels = pre-exercise and at 3, 6, 9, 12, and 15 min) and protocol (3 levels). Peak and mean values of these outcomes across protocols were compared using one-way ANOVA. If a significant F ratio was obtained, dependent t-test was used to detect differences between means. Effect sizes for ANOVA were identified by η^2^ with the following criteria: 0.02–0.13: small; 0.14–0.26: medium; and > 0.26: large. Cohen’s d was used as an estimate of effect size, with scores between 0.15 and < 0.40 representing “small, ” between 0.40 and < 0.75 representing “moderate, ” and ≥ 0.75 representing “large.” Our sample size is similar to or larger than prior studies assessing changes in SV and CO (Zafeiridis et al., 2015; Falz et al., 2019; Balci et al., 2022). Statistical significance was set at p < 0.05.

The sample size was estimated for CO with the following input parameters in G*Power version 3.1.9.7 (Dusseldorf University, Dusseldorf, Germany): (a) F test for one group and three measurements; (b) effect size of 0.35; (c) alpha-value of 0.05; (d) statistical power of 0.80; and (e) correlation between measures of 0.50. The calculated sample size was 15, which is lower than the actual sample size equal to 18.

## 3 Results

Maximal exercise responses: **Table 2** demonstrates outcomes acquired from VO_2_max testing. Participant VO_2_max values are classified in the 50 - 60^th^ percentile for men undergoing incremental cycling (Kaminsky et al., 2017). VO_2_max, Wmax, VT1, and maximal values of SV/CO reflect the heterogeneity of our participants.

**Table 2:** Gas exchange, hemodynamic, and metabolic results from participants undergoing VO_2_max testing.

Outcome	Mean ± SD	Range
VO_2_max (mL/kg/min)	41.4 ± 9.2	29.0 – 63.8
VO_2_max (L/min)	3.3 ± 0.7	2.3 – 4.9
V_E_max (L/min)	140 ± 29	88 - 185
RERmax	1.29 ± 0.09	1.12 – 1.46
Wmax (W)	305 ± 57	218 - 457
Wmax (W/kg)	3.9 ± 0.8	2.9 – 5.9
HRmax (b/min)	184 ± 8	166 - 197
SVmax (mL)	126 ± 20	87 - 164
COmax (L/min)	23.2 ± 3.6	16.2 – 29.5
a-vO_2_max (mL/dL)	14.2 ± 2.1	11.0 – 17.0
VT1 (%VO_2_max)	70.8 ± 5.1	64.2 – 81.7
VT1 (%Wmax)	64.2 ± 6.5	50.6 – 76.8
VT1 (%HRmax)	82.4 ± 5.1	73.0 – 89.0
BLa (mM)	11.4 ± 2.5	8.6 – 16.6

VO2max, maximal oxygen uptake; VE, ventilation; RER, respiratory exchange ratio; Wmax, maximal workload from VO2max test; HR, heart rate; SV, stroke volume; CO, cardiac output; a-vO2, arteriovenous oxygen difference; VT, first ventilatory threshold; BLa, blood lactate concentration.

Differences in %Wmax, gas exchange data, and BLa between MICT, VICT, and HIIT: These protocols elicited significantly different %Wmax (p < 0.001, η^2^ = 0.98), with *post hoc* analyses showing significant differences between means (all p < 0.001, d = 2.68 – 3.78). All results showing differences in mean/peak HR/V_E_/VO_2_, RER, and BLa between protocols are shown in Supplemental content (**Supplementary Results**; **Table 3**).

**Table 3:** Differences in Wmax, time > 77%HRmax, and mean and peak HR and VO_2_ between MICT, VICT, and HIIT (mean ± SD).

Outcome	MICT	VICT	HIIT
Wmax (%)	45 ± 5^a^	58 ± 6^a^	84 ± 8^a^
Time > 77%HRmax (min)	2.6 ± 4.0	11.2 ± 2.8^b^	11.1 ± 1.7^b^
Mean HR (b/min)	133 ± 13	154 ± 11^b^	155 ± 10^b^
Peak HR (b/min)	143 ± 14	170 ± 13^b^	173 ± 13^b^
Mean HR (%HRmax)	72 ± 6^a^	84 ± 4^a^	88 ± 7^a^
Peak HR (%HRmax)	78 ± 6	93 ± 5^b^	90 ± 6^b^
Mean VO_2_ (L/min)	1.93 ± 0.40^a^	2.43 ± 0.52^a^	2.25 ± 0.39^a^
Peak VO_2_ (L/min)	2.06 ± 0.40	2.68 ± 0.57^b^	2.59 ± 0.48^b^
Mean VO_2_ (%VO_2_max)	58.7 ± 3.9^a^	73.7 ± 5.8^a^	69.3 ± 7.3^a^
Peak VO_2_ (%VO_2_max)	63.0 ± 4.7	81.5 ± 6.8^b^	80.0 ± 8.4^b^
BLa (mM)	3.0 ± 0.9^a^	6.2 ± 1.8^a^	8.0 ± 2.2^a^
V_E_ (L/min)	54 ± 10	84 ± 23^b^	86 ± 18^b^
RER	0.96 ± 0.06^a^	1.01 ± 0.04^a^	1.05 ± 0.05^a^
EE (kcal)	145 ± 29^a^	182 ± 42^a^	171 ± 33^a^

Wmax, maximal workload; HRmax, maximal heart rate; HR, heart rate; VO2, oxygen uptake; MICT, moderate intensity continuous training; VICT, vigorous intensity continuous training; HIIT, high intensity interval training; VO2max, maximal oxygen uptake; BLa, blood lactate accumulation; VE, end-exercise ventilation; RER, end-exercise respiratory exchange ratio; EE, energy expenditure; ^a^p < 0.05 between all values; ^b^p < 0.05 vs. MICT.

Differences in the hemodynamic and avO_2_diff response between MICT, VICT, and HIIT: There was a significant main effect of protocol on mean CO (L/min) (p < 0.001, η^2^ = 0.77), with *post hoc* analyses showing significantly higher values in response to VICT (p < 0.001, d = 1.29) and HIIT (p < 0.001, d = 1.60) versus MICT. (**Table 4**). Similar trends were shown for peak CO (L/min) (p < 0.001, η^2^ = 0.74). Results exhibited no significant differences in mean (p = 0.17, η^2^ = 0.10) or peak SV (p = 0.31, η^2^ = 0.07) between protocols. There was a significant difference in mean avO_2_diff across protocols (p < 0.001, η^2^ = 0.37), with *post hoc* analyses revealing a significantly higher value in response to VICT compared to MICT (p = 0.009, d = 0.40) and HIIT (p < 0.001, d = 0.57). Similar differences across protocols were shown for peak avO_2_diff (p = 0.013, η^2^ = 0.23), with VICT exhibiting significantly higher values versus MICT (p = 0.01, d = 0.48) and HIIT (p = 0.01, d = 0.37).

**Table 4:** Mean and peak cardiac output, stroke volume, and arteriovenous oxygen difference between MICT, VICT, and HIIT (mean ± SD).

Outcome	MICT	VICT	HIIT
Mean SV (mL/beat)	124.9 ± 14.1	126.2 ± 16.8	128.5 ± 15.7
Peak SV (mL/beat)	128.0 ± 14.1	130.5 ± 17.5	132.2 ± 17.3
Mean CO (L/min)	16.7 ± 2.4	19.9 ± 2.7^a^	20.5 ± 2.5^a^
Peak CO (L/min)	18.2 ± 2.4	22.0 ± 2.8^a^	22.2 ± 2.9^a^
Mean avO_2_diff (mL/dL)	11.7 ± 1.7^b^	12.4 ± 2.0	11.4 ± 1.6^b^
Peak avO_2_diff (mL/dL)	11.6 ± 1.6^b^	12.5 ± 2.2	11.8 ± 1.7^b^

MICT, moderate intensity continuous training; VICT, vigorous intensity continuous training; HIIT, high intensity interval training; SV, stroke volume; CO, cardiac output; avO2diff, arteriovenous oxygen difference; ^a^p < 0.05 versus MICT; ^b^p < 0.05 versus VICT.

**Figure 1:**
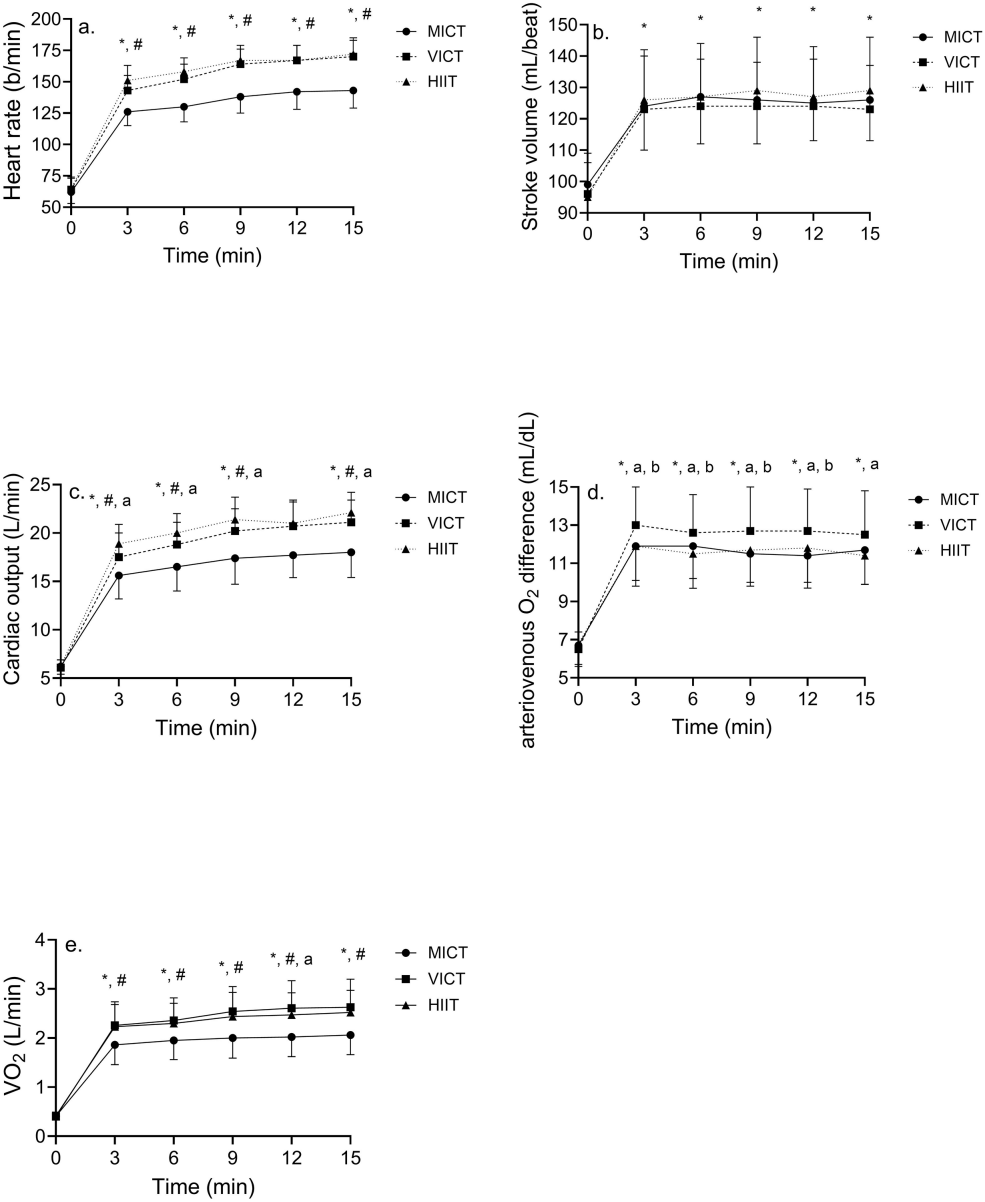
Differences in **(a)** heart rate; **(b)** stroke volume; **(c)** cardiac output, **(d)** arteriovenous oxygen difference, and **(e)** oxygen consumption between MICT, VICT, and HIIT. *p < 0.05 versus pre-exercise; ^#^p < 0.05 for VICT and HIIT versus MICT at same time point; ^a^p < 0.05 for HIIT versus VICT at same time point; ^b^p < 0.05 for VICT versus MICT at same time point; all data are expressed as mean ± SD.

**Figure 1** demonstrates changes in HR, SV, CO, avO_2_diff, and VO_2_ during exercise. HR significantly increased across time (p < 0.001, η^2^ = 0.98) and there was a significant effect of protocol (p < 0.001, η^2^ = 0.80) and protocol X time interaction (p < 0.001, η^2^ = 0.68) (**Figure 1a**). Compared to pre-exercise (64 ± 8 b/min), HR increased by 80, 106, and 108 b/min, respectively, in response to MICT, VICT, and HIIT, and *post hoc* analyses showed significant differences (p < 0.001) in all exercise HR values versus pre-exercise for all protocols (d = 7.0, 9.9, and 10.3, respectively). In addition, all HR values for all protocols were significantly different from each other (p < 0.001, d = 0.66 – 1.54), with exception of values between 9 and 12 (p > 0.09) and 12 and 15 min of exercise (p > 0.21). During exercise, VICT and HIIT revealed significantly higher (p < 0.001, d = 1.9 - 2.3) HR at all time points versus MICT, yet there was no difference in HR between VICT and HIIT (p > 0.10).

Stroke volume significantly increased during exercise by approximately 25–30 mL versus pre-exercise (97 ± 12 mL, p < 0.001, η^2^ = 0.90), yet there was no effect of protocol (p = 0.30, η^2^ = 0.07) or protocol X time interaction (p = 0.15, η^2^ = 0.08) (**Figure 1b**). *Post hoc* analyses showed that for all protocols, all exercise values were higher than pre-exercise (d = 2.4 – 2.6, p < 0.001) but there was no change in SV during exercise (p > 0.63).

Results showed a significant main effect of time for CO (p < 0.001, η^2^ = 0.97) as well as a significant effect of protocol (p < 0.001, η^2^ = 0.81) and protocol X time interaction (p < 0.001, η^2^ = 0.52) (**Figure 1c**). Pre-exercise CO was equal to 6.1 ± 0.7 L/min and significantly increased by 15 min of exercise to 18.0 ± 2.6 (p < 0.001, d = 6.2), 21.1 ± 2.3 (p < 0.001, d = 9.1), and 22.2 ± 2.2 L/min (p < 0.001, d = 9.7) in response to MICT, VICT, and HIIT, respectively. *Post hoc* analyses showed that across protocols, all exercise CO values were significantly different from each other (p < 0.004) except between 12 and 15 min (p > 0.21). VICT and HIIT exhibited significantly higher CO than MICT (p < 0.001, d = 1.0 – 1.9), and at 3, 6, 9, and 15 min, CO values were significantly higher (p < 0.042) in response to HIIT versus VICT (d = 0.50 – 0.64).

AvO_2_diff significantly increased from baseline to exercise (p < 0.001, η^2^ = 0.91) and there was a significant effect of protocol (p = 0.001, η^2^ = 0.34) and protocol X time interaction (p < 0.001, η^2^ = 0.22) (**Figure 1d**). AvO_2_diff remained constant during exercise for all protocols, yet *post hoc* analyses showed that all exercise values were significantly different (p < 0.001) compared to the pre-exercise value (d = 3.6 – 3.9). In addition, VICT showed significantly higher avO_2_diff compared to HIIT at all timepoints (p = 0.045 – 0.017, d = 0.43 - 0.61), with higher values observed during VICT versus MICT (p = 0.009 - 0.001, d = 0.40 – 0.60) with exception of avO_2_diff at 15 min of exercise (p = 0.68).

*Factors explaining the increase in exercise VO_2_*: Compared to baseline (0.41 ± 0.06 L/min), there was a substantial increase in VO_2_ from 1 to 15 min of exercise equal to 0.51 ± 0.23, 0.91 ± 0.26, and 0.70 ± 0.20 L/min for MICT, VICT, and HIIT, respectively (**Figure 1e**). There was a significant protocol X time interaction (p < 0.001, η^2^ = 0.65) and significant effect of time (p < 0.001, η^2^ = 0.95) and protocol (p < 0.001, η^2^ = 0.83). *Post hoc* results showed that all VO_2_ values were higher in response to VICT and HIIT versus MICT (p < 0.001, d = 0.89 – 1.59) and there were no differences in VO_2_ between VICT and HIIT, other than at 12 min of exercise (p = 0.03, d = 0.34). Results exhibit that this increase in VO_2_ was due to an increase in CO rather than an increase in avO_2_diff, which did not change during exercise. HR increased during exercise by 31 ± 10, 45 ± 12, and 42 ± 14 b/min for MICT, VICT, and HIIT, yet SV was maintained.

**Figure 2:**
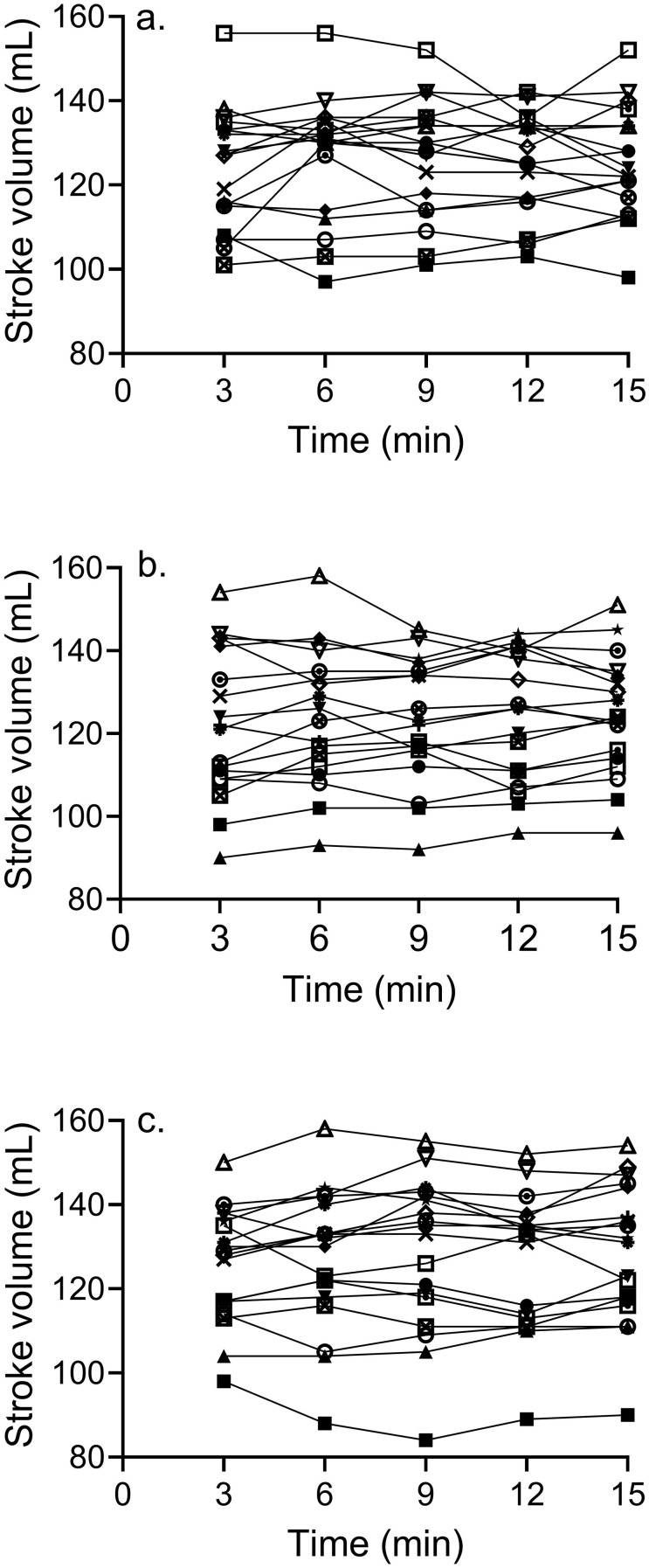
The stroke volume response to exercise in all participants (N = 18) for **(a)** MICT, **(b)** VICT, and **(c)** HIIT .

**Figure 2** exhibits varied SV responses across participants. For MICT, participants showed an increase (n = 5), decrease (n = 4), and no change in SV (n = 5) during exercise, and four participants showed an early increase followed by a decrease in SV in the last 3 min of exercise. During VICT, six participants showed an increase in SV, three showed a decrease, seven men showed no change in SV, and two showed an increase followed by a decline in SV. Similar responses were shown for HIIT, with seven, two, and six participants showing an increase, decrease, and no change in SV during exercise, and three showing an increase in SV followed by a decrease near the end of exercise.

## 4 Discussion

The present study compared hemodynamic responses between duration-matched sessions of MICT, HIIT, and VICT. Exercise intensity was prescribed relative to VT1 rather than %HRmax or %Wmax to provide greater physiological standardization across participants. However, because RCP was not determined, some participants may have exercised in different physiological domains during HIIT and VICT, which represents a limitation. The three exercise protocols were matched for duration but not for total energy expenditure, which should be considered when interpreting differences between conditions. Data show that VICT elicited the highest mean VO_2_ which was mediated by a significantly higher avO_2_diff, as there was no difference in CO between HIIT and VICT. There was no significant difference in SV during exercise and between protocols, yet varied responses were shown across participants. In addition, VICT and HIIT demonstrate a similar mean HR and time > 77%HRmax despite lower %Wmax in VICT. Overall, VICT and HIIT elicited similar HR and CO responses despite marked differences in exercise structure and workload. However, VICT elicited higher mean VO_2_ and avO_2_ diff, suggesting greater peripheral oxygen extraction during exercise.

In the present study, we used a rapid incremental protocol to determine VO_2_max and VT1, with VT1 used to prescribe intensities for subsequent sessions. Prior data show that a protocol having 1 min steps elicits similar VO_2_max as protocols having longer steps (2 or 3 min) (Amann et al., 2004; Roffey et al., 2007) yet leads to incidence of the VT at a significantly higher absolute intensity (Bentley et al., 2007; Roffey et al., 2007; **Table 2**). This is due to the lack of a steady-state during our rapid ramp protocol and in turn, accelerated metabolic and cardiovascular stress imposed on the participant. Consequently, it is possible that VT1-derived workloads were prescribed at higher absolute intensities than would occur with MRT-corrected thresholds, contributing to the elevated BLa responses observed during exercise. Ultimately, scientists need to consider the stage duration selected when using data from ramp protocols to prescribe exercise according to VT1. In addition, various physiological responses (%VO_2_, VT1, BLa, etc.) to subsequent exercise sessions cannot be compared across studies using ramp protocols having dissimilar work rate increments.

Prior studies show that attaining a high %VO_2_max during individual training sessions is related to long-term VO_2_max improvements (Buchheit and Laursen, 2013; Fleckenstein et al., 2025), while low-volume HIIT sessions (≤ 15 min) are sufficient to elicit favorable changes in cardiometabolic health (Yin et al., 2024). Our results show that both low-volume VICT and HIIT elicit vigorous exercise represented by substantial duration > 77%HRmax and peak intensities ≥ 80%VO_2_max and 90%HRmax (**Table 3**), although mean VO_2_ was 8% higher in response to VICT. The Fick equation states that this can be achieved by attaining high fractions of maximal CO and/or avO_2_diff. Our participants revealed mean and peak CO equal to ~85 and 96% maximal values in response to VICT and HIIT, with these values significantly higher than induced by MICT (mean and peak CO = 72 and 78% maximum). Results from active men and women (Coe and Astorino, 2024) reported similar mean and peak CO between 10 X 1 min HIIT at 85%Wmax and 4 X 4 min HIIT at 85 – 95%HRmax, supporting our data. In active men with VO_2_max = 54 mL/kg/min, CO attained approximately 70 – 90% maximum in response to repeated 3 min HIIT efforts at 80%Wmax (Ksoll et al., 2021). In response to VICT at 88%Wmax leading to exercise termination in 12 min, Lepretre et al. (2004) reported that CO peaked at 86% of maximal values. Overall, these results demonstrate that both HIIT and VICT elicit near-maximal CO responses despite substantial differences in exercise structure and absolute workload. Although speculative, this high CO is likely due to substantial drift in HR during constant load intensities above lactate threshold characteristic of VICT. Moreover, VICT exhibited a dramatic increase in VO_2_ equal to 0.91 L/min during exercise, which was significantly higher than HIIT, which would require a sizable increase in CO similar to HIIT. Furthermore, the intermittent structure of HIIT may reduce mean VO_2_ relative to continuous exercise because of delayed VO_2_ kinetics during repeated short intervals.

Our exercise SV values equal to 125–132 mL are comparable to those reported in similarly trained adults (Zafeiridis et al., 2015; Coe and Astorino, 2024), although lower than values (143–153 mL) reported by Lepretre et al. (2004) in triathletes with VO_2_max = 63 ml/kg/min. Higher SV values reported in endurance-trained adults are likely related to greater blood volume and enhanced ventricular filling capacity (Gledhill et al., 1994; Heinicke et al., 2001). Our results also showed no significant difference in mean and peak SV or the SV response across protocols (**Table 4**; **Figure 1**), in which SV rapidly increased from pre-exercise to 3 min after which it remained constant until end-exercise. This supports data from Lepretre et al. (2004) during 12 min of VICT at 88%Wmax. Falz et al. (2019) also showed no difference in mean SV between HIIT (4 X 4 min at 85 – 95%HRmax) and MICT (25 min at 70%HRmax) in active men. Lastly, exercise SV values were comparable to SVmax achieved from incremental exercise, suggesting that exercise at intensities ranging from 45 - 84%Wmax, which elicit varying HR responses, induce maximal SV, which may be important for increasing VO_2_max in response to exercise training (Lepretre et al., 2004; Helgerud et al., 2007).

Vella and Robergs (2005) documented four distinct patterns in SV during graded exercise, which included a linear increase in SV, a maintenance in SV, and a maintenance in SV with an increase or decrease occurring near VO_2_max. These unique patterns were also exhibited in women with diverse VO_2_max (Schierbauer et al., 2022) and results from Astrand et al. (1964) in men and women undergoing progressive exercise to VO_2_max. Several studies using different hemodynamic methods report consistent increases in SV during graded exercise in trained athletes (Zhou et al., 2001; Warburton et al., 2002) healthy women (Schierbauer et al., 2022), young men with no history of exercise training (Martino et al., 2002), and moderate and elite trained women (Wang et al., 2012). However, in healthy untrained men performing graded exercise, Higgenbotham et al. (1986) demonstrated no increase in SV above low intensities, supporting early data from Rushmer (1959). Our results show maintenance in SV during exercise and no significant difference across protocols. This is likely explained by the minimum intensity equal to 59 – 74%VO_2_max which exceeds the value at which SV may plateau (40 – 50%VO_2_max, Astrand et al., 1964). Nevertheless, our participants showed varying responses to exercise (**Figure 2**). Substantial inter-individual variability in SV responses was observed across all exercise conditions, consistent with previous reports using graded exercise protocols. A lack of change or small decrease in SV approaching VO_2_max has been explained by a regulatory limitation on the heart (Warburton and Gledhill, 2008); whereas, the increase in SV may be due to greater diastolic function (Zhou et al., 2001).

Our results show maintenance of avO_2_diff during exercise which support some prior data from continuous exercise. Vella et al. (2011) required active lean and obese adults to perform 20 min of cycling at 65%VO_2_max, and there was no change in avO_2_diff during exercise. However, Kawaguchi et al. (2006) demonstrated a significant increase in avO_2_diff during cycling at 50%VO_2_max. In contrast, fatiguing exercise at 88 and 100%Wmax (duration = 12.1 and 5.2 min) led to a significant decrease in avO_2_diff which was attendant with significant desaturation (Lepretre et al., 2004). These discrepant results across studies may be due to different participants recruited, intensity and duration of the exercise completed, and method of CO/avO_2_diff assessment (thoracic impedance versus Modelflow). VICT revealed a significantly higher avO_2_diff versus HIIT throughout exercise, a novel result, which may have enabled a significantly higher mean VO_2_ (8%) and slightly higher peak VO_2_ (4%). Further research using more direct measures is needed to confirm this result and ultimately, better understand factors mediating higher avO_2_diff and VO_2_ induced by VICT.

Several limitations should be considered when interpreting these findings. First, data were acquired in healthy young men, so results cannot be generalized to women and unhealthy adults, although Coe and Astorino (2024) showed no differences in hemodynamic responses between men and women undergoing three different HIIT protocols. Men had disparate values of BMI and body mass, which may lead to unique cardiovascular responses to the three exercise protocols. In addition, these results should be confirmed using longer duration MICT, higher volume HIIT regimens such as the 4X4 HIIT protocol, and low volume exercise such as sprint interval training. Significantly higher values of SV and CO would be revealed if these protocols were performed on the treadmill, due to the greater VO_2_max elicited by whole-body exercise compared to cycling (Hermansen et al., 1970). Although thoracic impedance provides continuous estimates of stroke volume and cardiac output during exercise and has demonstrated acceptable reliability in previous studies using vigorous exercise (Gordon et al., 2018) and up to VO_2_max (Richard et al., 2001), its accuracy during vigorous exercise, when blood volume is reduced, remains debated (Cheung et al., 2020). Accordingly, our findings should be interpreted with appropriate caution and confirmed using alternative methodologies. Yet, the study is strengthened by use of a large, heterogeneous sample and acquisition of hemodynamic responses using thoracic impedance which provides continuous assessment of SV and CO rather than singular values acquired using other methods, such as acetylene rebreathing (McCole et al., 2001). Lastly, this novel study elucidates changes in SV, CO, and avO_2_diff to different types of exercise (MICT, VICT, and HIIT) which better represent typical exercise patterns in adults. As noted above, the majority of data describing the hemodynamic response to exercise are derived from incremental rather than continuous exercise.

Overall, VICT elicited significantly higher mean VO_2_ and avO_2_diff compared with HIIT despite similar CO responses, consistent with a greater contribution of peripheral oxygen extraction during continuous vigorous exercise. Both HIIT and VICT induced substantial cardiovascular stress and near-maximal CO responses despite markedly different exercise structures. Stroke volume responses varied considerably across participants, supporting previous findings demonstrating heterogeneous cardiovascular responses during incremental exercise. Future longitudinal studies are needed to determine whether these acute physiological differences translate into distinct long-term cardiovascular adaptations.

## Data Availability

The original contributions presented in the study are included in the article/**Supplementary Material**. Further inquiries can be directed to the corresponding author.
