# Supplementary material for: Acute hemodynamic responses to high intensity interval training, moderate intensity continuous training, and vigorous intensity continuous training in active men
Source: Front Physiol. 2026 Jul 1;17:1832895. doi: 10.3389/fphys.2026.1832895 (PMC13368661; doi:10.3389/fphys.2026.1832895)
Supplement: Supplementary file 1 [file Table1.docx]

**Supplemental Results**

These results are shown in Table 3. There was a significant difference in time > 77 %HRmax across protocols (p < 0.001, η^2^ = 0.81), with post hoc analyses showing significant differences (p < 0.001) between VICT and HIIT versus MICT (d = 2.59 and 2.82). Similar results across protocols were shown for mean/peak HR expressed as b/min and %HRmax (p < 0.001, η^2^ = 0.78 – 0.85), yet for mean HR (%HRmax), all means were significantly different from each other (p < 0.01). Results showed a significant effect (p < 0.001, η^2^ = 0.78) of protocol on mean VO_2_ (L/min and %VO_2_max), and post hoc analyses showed that all means were significantly different from each other, with HIIT (p < 0.001, d = 1.8) and VICT (p < 0.001, d = 2.5) exhibiting significantly higher values compared to MICT, and VICT eliciting higher mean VO_2_ versus HIIT (p = 0.016, d = 0.69). In regards to peak VO_2_ (L/min and %VO_2_max), there was a main effect of protocol (p < 0.001, η^2^ = 0.83) and post hoc results showed significantly higher values in response to VICT and HIIT versus MICT (d = 2.62 and 3.18).

There were significant differences in BLa (p < 0.001, η^2^ = 0.79), V_E_ (p < 0.001, η^2^ = 0.71), RER (p < 0.001, η^2^ = 0.62), and EE (p < 0.001, η^2^ = 0.78) across protocols. All BLa values were significantly different (p < 0.001) across protocols, with the highest value demonstrated in HIIT versus VICT and MICT (d = 0.90 and 3.06), respectively. Post hoc analyses showed significantly higher (p < 0.001) end-exercise V_E_ for VICT (d = 1.8) and HIIT (d = 2.1) compared to MICT. Results showed that MICT yielded the lowest RER (p < 0.001 across all protocols) versus VICT (d = 0.95) and HIIT (d = 1.65), with RER being significantly higher in response to HIIT compared to VICT (d = 0.91). VICT (d = 1.05) and HIIT (d = 0.89) revealed significantly higher (p < 0.001) EE compared to MICT, with EE from VICT being significantly higher than HIIT (p = 0.025, d = 0.36). Enjoyment was significantly different across protocols (p = 0.03, η^2^ = 0.66), and post hoc analyses showed significantly greater enjoyment in response to HIIT (103 ± 13) versus MICT (93 ± 23, p = 0.04, d = 0.55) and VICT (93 ± 16, p = 0.002, d = 0.71).
